# Strain Sensor of Carbon Nanotubes in Microscale: From Model to Metrology

**DOI:** 10.1155/2014/406154

**Published:** 2014-02-10

**Authors:** Wei Qiu, Shi-Lei Li, Wei-lin Deng, Di Gao, Yi-Lan Kang

**Affiliations:** Tianjin Key Laboratory of Modern Engineering Mechanics, Department of Mechanics, Tianjin University, Tianjin 300072, China

## Abstract

A strain sensor composed of carbon nanotubes with Raman spectroscopy can achieve measurement of the three in-plane strain components in microscale. Based on previous work on the mathematic model of carbon nanotube strain sensors, this paper presents a detailed study on the optimization, diversification, and standardization of a CNT strain sensor from the viewpoint of metrology. A new miniaccessory for polarization control is designed, and two different preparing methods for CNT films as sensing media are introduced to provide diversified choices for applications. Then, the standard procedure of creating CNT strain sensors is proposed. Application experiments confirmed the effectiveness of the above improvement, which is helpful in developing this method for convenient metrology.

## 1. Introduction

The requirement of mechanical measurement is increasing with the rapid development of micro-nanoscience and technology [1]. Owing to its peculiarities, such as nondestructiveness, convenience, and high-resolution, micro-Raman spectroscopy has been widely applied to on-line in situ measurement of residual stress in microstructure caused by the processing [2, 3] and to real-time mechanical investigation of graphene, carbon nanotubes, and other low-dimensional materials under force loading [4–7].

Mechanical measurement based on Raman spectroscopy has a kernel limitation, for it is only applicable to “Raman-active” materials. This could be overcome by using “Raman-active” materials as sensors or sensing media. Carbon nanotubes (CNT) may become robust sensors in mechanical measurement by Raman because of their excellent mechanical and spectral properties [8, 9]. The Raman-strain sensitivity of CNTs was investigated by Cronin et al. [10] using atomic force probe to apply axial strain on single-walled carbon nanotube and recording the Raman peak position change with strain. Zhao and Wagner et al. [11–15] introduced the idea of a CNT strain sensor by Raman. Qiu et al. [16] presented a mathematical model for the measurement of all the in-plane strain components (normal strain, *ε*_*X*_, *ε*_*Y*_, and shear strain, *γ*_*XY*_) by utilizing polarized Raman and evaluating the quantitative contributions of carbon nanotubes in all directions to the entire spectrum. By using this model and Raman strain rosette technology that is proposed, the three in-plane strain components can be achieved in microscale by measuring the Raman information of CNTs randomly distributed on the measured sample with three specified polarization directions [17, 18].

The interface between CNTs and the matrix (bonding or attaching CNTs on the sample surface) behaves as a primary influence on the sensing properties, such as measuring range, sensitivity, and stability, of the CNT strain sensor. It is not only the materials but also the methods of preparing CNTs or their composite films that influence sensing properties on samples. The experiments by Schadler et al. [19] proved that multiwalled carbon nanotubes (MWNTs) were not suitable for strain sensing due to the low interface load transfer efficiency. Ma et al. [20] studied the behavior of load transfer in functionalized single-walled carbon nanotube (SWNT)/epoxy nanocomposites and showed that some functionalized groups may effectively improve the interface efficiency. Ma and Larsen [21] presented a comparative study on dispersion and interfacial properties of three different modified single-walled carbon nanotubes and three different polymer (PC, PVDF, and Epoxy) composites using Hansen solubility parameters and found that the association of the −COOH functionalized SWNT and epoxy had the highest interface load transfer efficiency. In addition to the sensing media, it has been proved that the method control of polarization direction in the Raman system also affects the precision of a CNT strain sensor [22].

Therefore, the equipment, materials, and procedure of this method should be optimized to improve the sensing properties and then diversified and standardized for convenience and efficiency. This work presents a detailed study on the optimization, diversification, and standardization of CNT strain sensors by Raman to promote development from a model to convenient metrology.

## 2. Mathematic Model of CNT Strain Sensor

The CNT strain sensor in microscale requires CNTs deforming together with the measured object and uses polarized micro-Raman spectroscopy to detect the strain information of the CNTs. For each individual CNT, its Raman G' peak (namely, Raman shift) changes coincident with the in-plane strain with small deformation,

(1) $$\begin{array}{c} \Delta \omega_{\left(\theta \right)}=\Psi_{\text{Sensor}}\cdot \left(\varepsilon_{X}\cos^{2}\theta +\varepsilon_{Y}\text{si}{\text{n}}^{2}\theta -\gamma_{xy}\cos\theta \sin\theta \right), \end{array}$$

where *θ* is the axial direction of the carbon nanotubes, Δ*ω* is the Raman shift increment after deformation, Ψ_Sensor_ is the strain-Raman shift coefficient of the CNT sensor, *ε*_*X*_, *ε*_*Y*_ and *γ*_*XY*_ are the normal strain components in the *X*, and *Y*-directions and the shear strain component of the in-plane strain, respectively.

For real measurement, it is more practical to use a CNT film, which contains a large number of randomly oriented carbon nanotubes, as the strain sensor rather than single CNTs. The film can be adhered to the surface of the measured object. In Raman measurement, the sampling point contains hundreds or thousands of carbon nanotubes in different directions. The spectrum information obtained by the microscopic Raman system is essentially the sum of all scattering information of CNTs within the sampling point.

Because the G' peak of CNTs is a Gauss (or Lorenz) shape, the properties of normal (or Cauchy) distribution are applied to the peak position of overall spectrum as follows [18]:

(2) $$\begin{array}{c} \Delta \Omega^{\left(\varphi \right)}=\frac{\int_{-\pi /2}^{\pi /2}\Delta \omega_{\left(\theta \right)}\cdot R\left(\theta -\varphi \right)\cdot \rho \left(\theta \right)d\theta }{\int_{-\pi /2}^{\pi /2}R\left(\theta -\varphi \right)\cdot \rho \left(\theta \right)d\theta }. \end{array}$$

The variable Δ*Ω* ^(*φ*)^ represents the Raman shift increment after deformation when the polarization angle of the incident light is *φ*.  *R*  is a function of the CNTs polarized Raman antenna effect, and *ρ*(*θ*) is the CNT plane distribution function. For the CNT film whose direction is randomly distributed, *ρ*(*θ*) is a constant. By substituting the detail function form of Δ*ω* and  *R*  in (3), the analytical relationship between the polarized Raman results and the in-plane strain components of the measured object was achieved, whose final form lies in the polarization configuration and control method of the Raman system. For instance, keeping the incident and scattering polarization direction parallel, *R*(*θ* − *φ*) = cos⁡^4^⁡(*θ* − *φ*), the relationship equation (2) becomes (3) [18],

(3) $$\begin{array}{c} \Delta \Omega^{\left(\varphi \right)}=\frac{1}{6}\Psi_{\text{Sensor}}\cdot {\left[\begin{array}{c} 3+2\cos2\varphi \\ 3-2\cos2\varphi \\ -2\sin2\varphi \end{array}\right]}^{Τ}\cdot \left[\begin{array}{c} \varepsilon_{X}\\ \varepsilon_{Y}\\ \gamma_{XY} \end{array}\right]. \end{array}$$

According to the above equation, the Raman shift increment at any polarization angle can be expressed by a linear combination of the in-plane strain components with different weights, either of which is a trigonometric function of polarization angle. Given three different polarization angles, such as 0°, 45°, and 90°, a simultaneous equation can be set up and the strain components solved,

(4) $$\begin{array}{c} \varepsilon_{X}=\frac{1}{4\Psi_{\text{Sensor}}}\cdot \left(5\Delta \Omega^{\left(0\right)}-\Delta \Omega^{\left(90\right)}\right),\\ \varepsilon_{Y}=\frac{1}{4\Psi_{\text{Sensor}}}\cdot \left(5\Delta \Omega^{\left(90\right)}-\Delta \Omega^{\left(0\right)}\right),\\ \gamma_{XY}=\frac{3}{2\Psi_{\text{Sensor}}}\cdot \left(\Delta \Omega^{\left(0\right)}+\Delta \Omega^{\left(90\right)}-2\Delta \Omega^{\left(45\right)}\right). \end{array}$$

## 3. Equipment, Material and Procedure of Metrology

### 3.1. Measurement System and Control Method

Strain measurement using a CNT sensor requires a research level cofocus micro-Raman spectrometer, such as the InVia series by Renishaw, LabRam HR series by Horiba JY, or Alpha series by Witec. In the Raman system, a He-Ne laser is preferred because the Raman spectra of metal SWNTs show outstanding antenna effects when excited by a 632.8 nm laser [23]. Moreover, according to (2), the detailed relationship between Raman shift and strain components depends on the polarization configuration and control method of the Raman system. Hence, the polarization of the Raman system should be controllable.

The control method of the current micro-Raman system requires special improvement for strain measurement using the CNT sensor. It has been shown that the dual coordination configuration, which regulates the incident and scattered polarization directions continuously and parallel permanently, is most suitable for a CNT strain sensor [22]. This configuration was traditionally realized by using a polarizer in the incident path and an analyzer in the scattering path. The incident polarizer is usually placed at the open space of the laser export, while the analyzer can be located inside the spectrometer and generally needs to be customized and processed by the user. Moreover, control of scattering polarization requires the user to repeatedly open the box of the spectrometer to regulate the analyzer.

The dual coordination configuration can also be achieved by a more practicable method as follows. A half-wave plate, whose frequency is similar to that of the Raman laser, replaces the polarized plate inside the 180° (or 360°) continuous polarizer, a standard accessory of the microscope in the Raman system. The transformed continuous polarizer is inserted in the open slot of the microscope when applying the CNT strain sensor. Meanwhile, the orthogonal analyzer, also a standard accessory of the Raman system, is used and adjusted to the parallel polarization direction.

**Figure 1:**
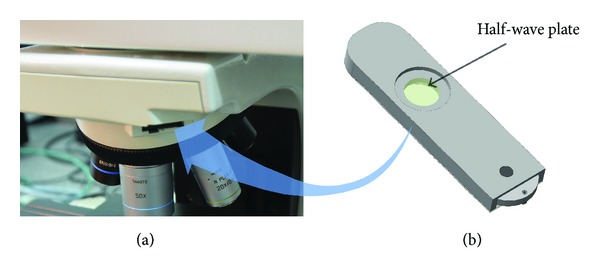
(a) 45° slot for the *λ*-plate of the microscope in the Raman system, (b) miniaturization-designed 180° continuous polarizer.

The above method has some exceptions, especially for Raman systems that lack open slots in the microscopes, use a UV laser, or lack dual-scan accessories, or other reasons. Even then, there is an alternative to the traditional method. As Figure 1 shows, the microscope in the Raman system always has a small 45° slot for a *λ*-plate, through which a miniature-designed 180° continuous polarizer (shown in Figure 1(b)) can be inserted into the public path of the incident and scattered light inside the microscope.

### 3.2. Preparing the Sensing Media

Single-walled carbon nanotubes (SWNTs) functionalized by a –COOH group (TIMESNANO Ltd.) were optimized as the “sensor” material. This is because the Raman spectra of SWNTs show an outstanding antenna effect [23], and the –COOH group improves the interface between the nanotubes and the polymer matrix [21]. Meanwhile, E51 epoxy resin, with low viscosity and room temperature curing, was selected as a polymer matrix, adhering the SWNTs onto the surface of the measured object.

Two methods are presented to prepare the CNT film onto the surface of the measured object, named the “ultrasonic blending method” and “coating-transfer method,” respectively. For the former one, 1 wt% SWNTs were mixed and dispersed into a liquid epoxy for 24 h using ultrasound in 60°C and then mixed with the curing agent (25 wt%) in 80°C. The mixture was dropped onto the surface of the measured object and then covered with a quartz glass plate coated by a release agent. The glass plate was pressed slowly, loaded by weight to extrude the CNT/epoxy mixture to a thin film, and then peeled from the sample carefully after curing the epoxy at 60°C for 5 h and at room temperature for 24 h.

The “coating-transfer method” was composed of two steps. For the first step, the SWNTs were dispersed in deionized water using ultrasound, then the liquid mixture film was coated on a polyethylene terephthalate (PET) plate by using a single roller-coating machine. When the water was completely volatilized, a pure CNT film was achieved. Next, the film was transferred onto the measured object by coating an epoxy film on the sample surface. The CNT film side of the PET plane was pressed on the surface slowly, then weights were loaded on the plane until the resin was totally cured, and the PET plane was peeled from the sample carefully.

### 3.3. Sensing Properties and Calibrations

For the study of the properties of CNT films as strain sensors, free-standing CNT film samples were prepared by using the above two methods and replacing the measured objects as quartz glass plates coated by release agents. After peeling the films from the glass plates, the films were cut to 50 × 3 mm^2^ strips. The strip samples were tagged as Film-I for those made by the ultrasonic blending method and as Film-II for those made by the coating-transfer method.

**Figure 2:**
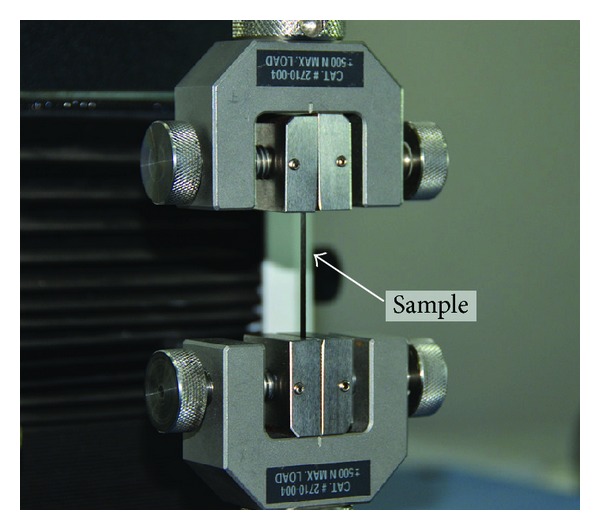
Sample under uniaxial tension.

**Figure 3:**
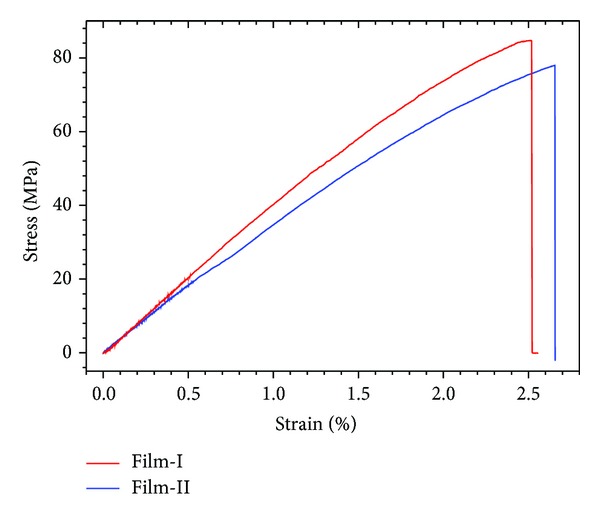
The typical stress-strain curves of the films under uniaxial tension.

The basic mechanical properties of the films were measured by utilizing an Instron 3343 testing machine (Figure 2). The loading rate was 0.05 mm/min. The typical stress-strain curves of the films under uniaxial stretching are shown in Figure 3. According to the experiment results, the mechanical properties of the Film-I and Film-II were almost similar. In detail, Young's modulus of Film-I and Film-II were 3.49 GPa and 4.13 GPa, tensile strengths were 77.96 MPa and 84.73 MPa, and the elongations were 2.66% and 2.56%, respectively.

The Raman-mechanical characteristics, namely, sensing properties, were achieved through calibration tests as follows. A Renishaw InVia Reflex Raman spectroscope with a He-Ne laser source (632.8 nm, 2 mW) was utilized, and the incident beam was focused on the CNT film surface of each sample in a backscattering geometry through a 50x objective lens (N.A. = 0.8), forming a sample spot of approximately 2 *μ*m in diameter. Each CNT film strip was uniaxially stretched step-by-step using a minitensile machine specially designed for the micro-Raman system. Before loading, 21 random points on the surface of each specimen were detected by the Raman system. At every point, Raman data with nine different polarization directions, from 0° to 90°, were achieved. Under each loading step, the Raman spectra around the G' band (2450 to 2800 cm^−1^) of the same sampling spot in 0°, 45°, and 90° polarization directions, were recorded. The G' peak of the spectra near 2650 cm^−1^ is fitted by using a Gauss or Lorenz function. Because the characteristic peaks of epoxy resin do not exist within the scope of 2450–2850 cm^−1^, the Raman shift of the G' peak is caused by deformation of carbon nanotubes.

**Figure 4:**
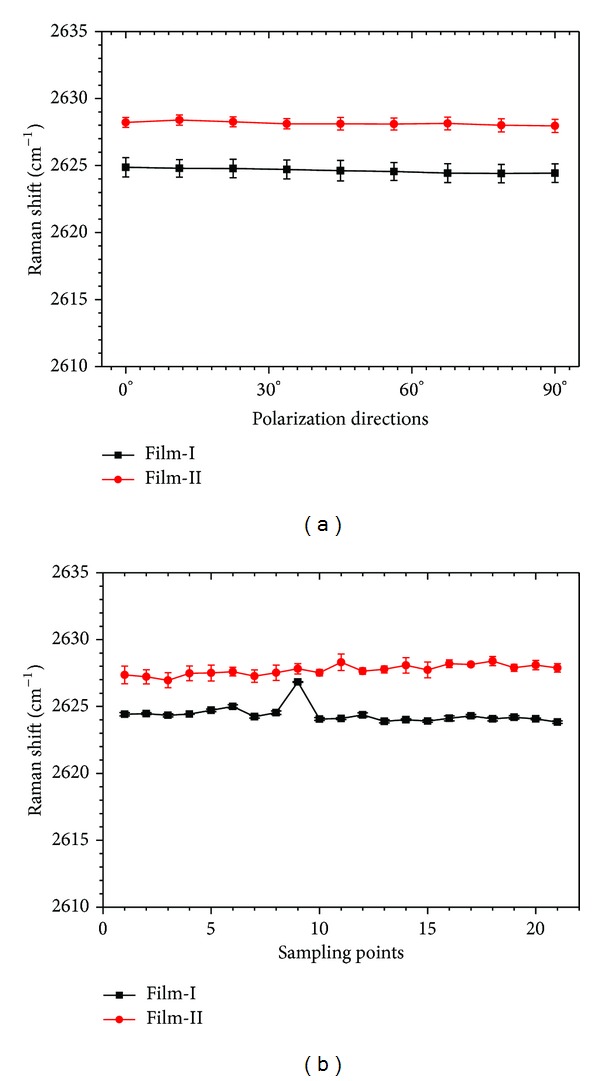
(a) Raman shifts (average of 21 points) at nine different polarization directions without loading. (b) Raman shifts (average of nine polarization directions) of 21 different points without loading.

Figures 4(a) and 4(b) give the Raman shift data of each film without loading. In Figure 4(a), the Raman shifts (average of 21 points) in nine different polarization directions are almost similar. In Figure 4(b), the Raman shifts (average of nine polarization directions) of 21 different points are also accordant. All these results show that the CNTs disperse uniformly inside both Film-I and Film-II.

**Figure 5:**
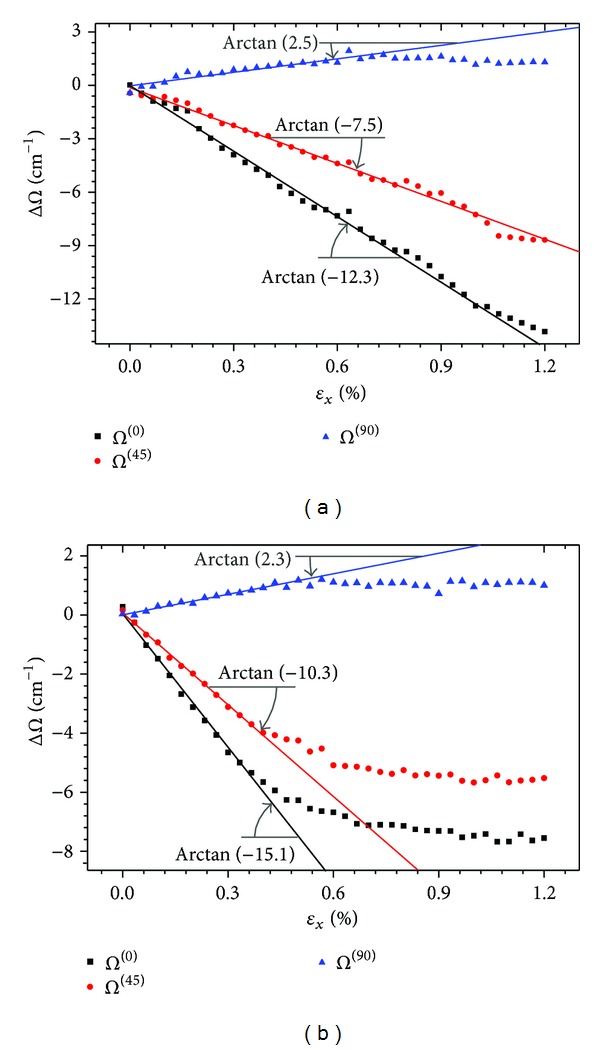
The results of the calibration experiments on (a) Film-I and (b) Film-II.

Figure 5(a) gives the results of the calibration experiment on Film-I, which show that the Raman shift change of polarization directions 0°, 45°, and 90° (ΔΩ^(0)^, ΔΩ^(45)^, and ΔΩ^(90)^) maintains satisfactory linearity in 1.0% strain extent. When  *ε*_*x*_  exceeds this extent, the Raman shift change becomes nonlinear. Hence, the segment inside this strain extent represents the measuring range of Film-I as strain sensor. Similarly, Figure 5(b) gives the results of the calibration experiment of Film-II, showing the measuring range of 0.4%.

The data in the linear segments were fitted (shown in Figure 5). Meanwhile, (5) was concluded by using the partial derivative of the first equation of (4),

(5) $$\begin{array}{c} \Psi_{\text{Sensor}}=\frac{1}{4}\cdot \left(5\frac{\partial \Delta \Omega^{\left(0\right)}}{\partial \varepsilon_{X}}-\frac{\partial \Delta \Omega^{\left(90\right)}}{\partial \varepsilon_{X}}\right). \end{array}$$

The fitted data in Figure 5 were substituted into (5), achieving the strain-Raman shift coefficients of each CNT film as a strain sensor. For the CNT film made by the ultrasonic blending method, Ψ_Sensor_ = − 1600 cm^−1^. Similarly, for the film made by the coating-transfer method, Ψ_Sensor_ = − 1945 cm^−1^.

There are obvious differences of the sensing properties (listed in Table 1) between two CNT films, which are mainly caused by the interfacial strength and rigidity between the carbon nanotubes and epoxy matrix. The CNT/epoxy interfaces in Film-I have higher average strength, showing a relative large measuring range. The interface in Film-II behaves more rigidly, corresponding to a bigger sensitivity. Such differences provide diversified choices for application.

### 3.4. Standard Procedure

**Figure 6:**
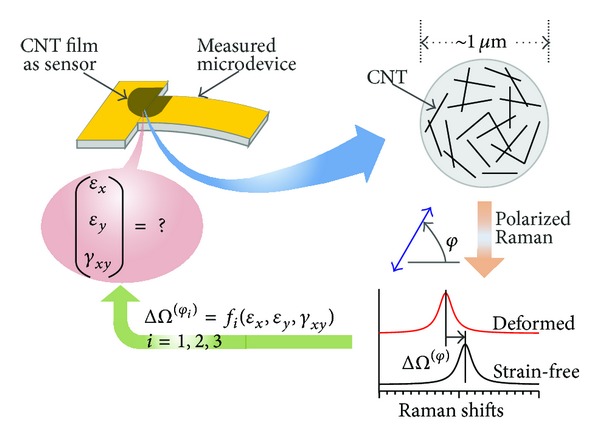
The standard procedure of strain measurements using the CNT strain sensor.

As shown in Figure 6, the standard procedure of strain measurements using a CNT strain sensor is as follows. A CNT film is prepared on the surface of the measured microdevice. The sample is put into the microscope of the cofocus micro-Raman spectrometer, whose polarization is controlled with the dual coordination configuration by using the transformed continuous polarizer. The laser is focused on the CNT film using a high magnification lens with a sampling spot of 1 to 2 *μ*m, where there exist thousands of randomly oriented CNT individuals. The Raman spectra of every sampling spot with different polarization angles, such as 0°, 45°, and 90°, are detected before and after deformation. The G' peak of the spectra near 2650 cm^−1^ are fitted by using a Gauss or Lorenz function. The Raman shift increments are substituted into (4); hence, all the strain components of the sampling spot are obtained. Through Raman mapping, the distribution field of each strain component may be achieved.

## 4. Applications and Discussions

**Figure 7:**
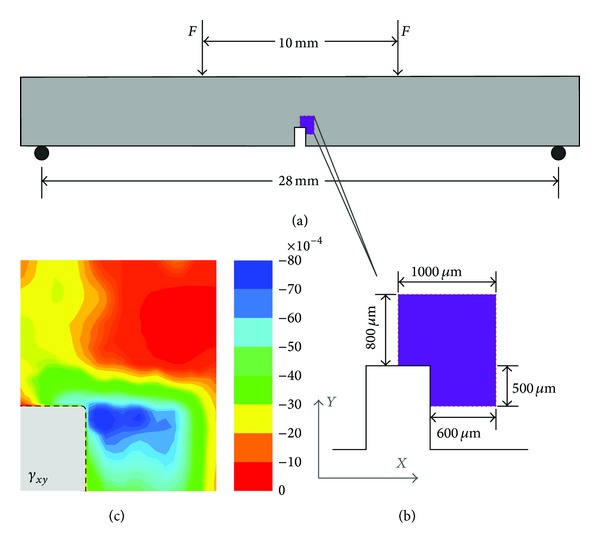
The experiment on the fiber-reinforced bar under four-point bend loading. (a) The geometrical shape, dimensions, and loading type, (b) Raman mapping region, (c) the distribution of  *γ*_*xy*_  near the vicinity of the slop tip achieved by the CNT strain sensor.

To prove the metrology advantage of the CNT sensor, a series of applications were performed on the samples.  Figure 7 shows four-point bend experiments on a fiber-reinforced epoxy bar (Figure 7(a)) whose dimensions are 32 × 6 × 2 mm^3^. The epoxy is DGEBA-based epoxy resin (E51), with Young's module of 3.5 GPa and a Poisson ratio of 0.3. The unidirectional carbon fiber (Toray M40JB-12k) is parallel to the length direction (namely, *x*-direction). The interface strength of resin and fiber is approximately 50–100 MPa. A slot, approximately 0.8 mm wide and 2 mm tall, normal to the *x*-direction, was prepared at the middle of one longitudinal side of the bar. Then, a CNT film that was 30 *μ*m thick was prepared on the epoxy bar surface. The sample was four-point bend loaded to a 300 *μ*m maximum deflection. An area next to the rectangle tip of the slot (shown in Figure 7(b)) was scanned with a 50 *μ*m step length by using the Raman spectroscope (Renishaw InVia Reflex, 632.8 nm laser, 50x lens) and applying the CNT strain sensor at each spot.

The experimental result of shear strain distribution is given in Figure 7(c), which shows that nonuniform deformation exists near the vicinity of the slop tip. Particularly, the shear zone is parallel to the fiber direction, neither the slope direction nor the 45° direction, which shows the specific mechanical behavior of unidirectional fiber-reinforced materials. Quantitatively, the maximum of the shear strain is approximately 8 × 10^−3^; thus, the shear stress reaches 10.8 Mpa, where *τ* = *Eγ*/2(1 + *μ*) = 3.5 × 8/2(1 + 0.3) = 10.8 MPa.

**Figure 8:**
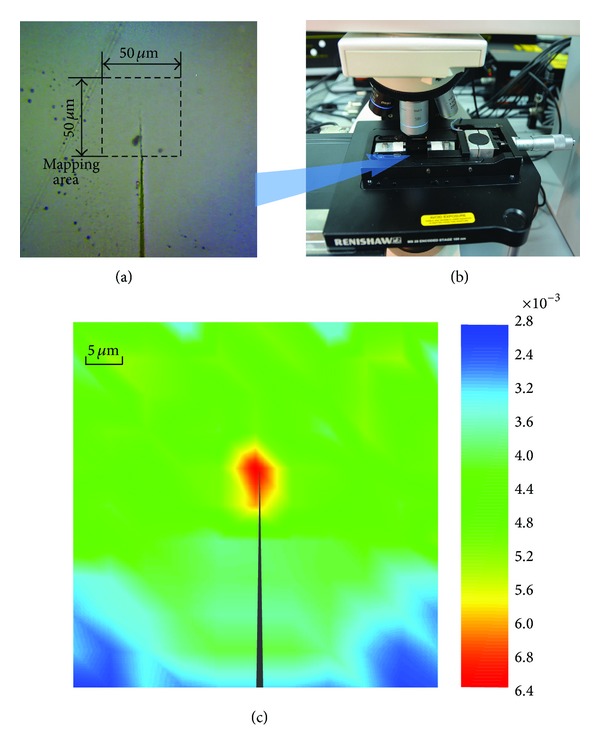
The experiment on the mode I crack. (a) Experimental system, (b) the image of the sample and Raman mapping region, (c) the distribution of  *ε*_*x*_  around the crack tip.

Figure 8 shows the experimental result of strain distribution near the tip of a mode I crack by using the CNT strain sensor. The sample was a CNT/epoxy composite made by ultrasonic blending method (Film-I), whose dimensions were 50 × 3 × 0.05 mm^3^. A transverse crack (Figure 8(a)), approximately 0.5 mm in length, was cut normal to the longitudinal direction of the sample by using a razor blade. The strip sample was uniaxially loaded to 0.3% tensile mean strain. The vicinity around the crack tip, 50 × 50 *μ*m^2^, was scanned with the Raman spectroscope (Renishaw InVia Reflex, 632.8 nm laser, 50x lens, shown in Figure 8(b)) in 5 *μ*m steps. Figure 8(c) gives the distribution of normal strain in the *x*-direction (via the tensile direction). It shows that  *ε*_*x*_  behaves as a serious concentration in front of the crack tip and even in the whole measured area.

**Figure 9:**
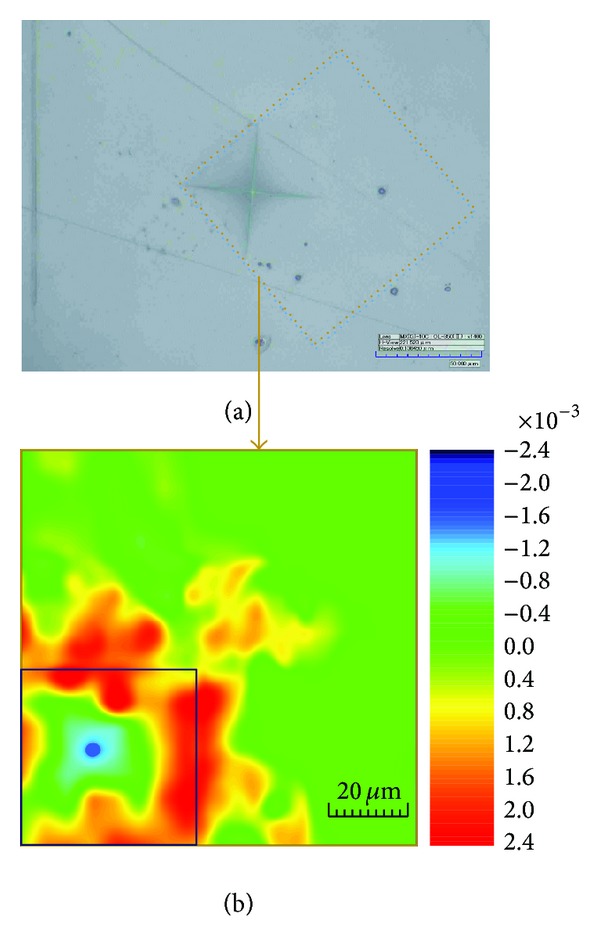
The experiment on the Vickers microindentation. (a) The image of the sample and Raman mapping region, (b) the distribution of residual in-plane principal strain (*ε*_*x*_ + *ε*_*y*_).

Figure 9 shows the experimental result of the residual strain field around a Vickers microindentation. The sample was a CNT/epoxy composite made by ultrasonic blending method (Film-I), whose thickness was also approximately 50 *μ*m. The sample was pressed by a Vickers microindenter gradually, until the force reached 0.5 N. After leaving the microindenter, the vicinity inside and outside the indentation, as shown in Figure 9(a), was scanned by the Renishaw InVia Reflex Raman spectroscope with a 632.8 nm laser and a 50x lens. Figure 9(b) gives the distribution of the sum of the in-plane principal strain ( = *ε*_*x*_ + *ε*_*y*_), which demonstrates that there are distinct residual compressive strains at the middle of the indentation and residual tensile strains at the edge of the indentation.

## 5. Conclusions

In this paper, the strain sensor composed of carbon nanotubes in microscale was studied, and several improvements were achieved. The applicable micro-Raman systems and the control modes of polarizations were discussed, and a new a miniaccessory for polarization control was designed. Then, two different preparing methods of CNT films as sensing media were presented. Calibration experiments showed that both preparing methods had high stabilities. The differences of sensing properties between the two methods provide diversified choices for application. Also, a standard procedure of measurement using the CNT strain sensor is proposed. Through several application experiments (such as vicinity round a notch area, mode I crack tip, and Vickers indentation), the effectiveness of the improvements was proved, promoting the strain sensor of carbon nanotubes from a model to very convenient metrology.

## Figures and Tables

**Table 1:** Strain-sensing properties of CNT films prepared by different methods.

CNT films	∂ΔΩ^(0)^/∂*ε*_*U*_	∂ΔΩ^(45)^/∂*ε*_*U*_	∂ΔΩ^(90)^/∂*ε*_*U*_	Ψ_Sensor_	Range
	(cm^−1^/%*ε*)	(cm^−1^/%*ε*)	(cm^−1^/%*ε*)	(cm^−1^/*ε*)	(*ε*)
Film-I	−12.3	−7.1	2.5	−1600	1.0%
Film-II	−15.1	−10.3	2.3	−1945	0.4%
